# Surveillance and Reduction Control of *Escherichia coli* and Diarrheagenic *E. coli* During the Pig Slaughtering Process in China

**DOI:** 10.3389/fvets.2021.735076

**Published:** 2021-10-05

**Authors:** Lin Wang, Na Liu, Yubin Gao, Junhui Liu, Xiumei Huang, Qingqing Zhang, Yuehua Li, Jianmei Zhao, Junwei Wang, Ge Zhao

**Affiliations:** China Animal Health and Epidemiology Center, Livestock and Poultry Products Quality and Safety Risk Assessment Laboratory of MARA, Qingdao, China

**Keywords:** *Escherichia coli*, diarrheagenic *E. coli*, pig, slaughtering progress, pre-cooling treatment, microbiological testing

## Abstract

The microbial contamination of pork during the slaughter process, especially that of the hygiene indicator bacteria, *Escherichia coli*, is closely related to the safety and quality of the meat. Some diarrheagenic *E. coli* can cause serious foodborne diseases, and pose a significant threat to human life and health. In order to ascertain the current status of *E. coli* and diarrheagenic *E. coli* contamination during the pig slaughter process in China, we conducted thorough monitoring of large-sized slaughterhouses, as well as small- or medium-sized slaughterhouses, in different provinces of China from 2019 to 2020. The overall positive rate of *E. coli* on the pork surface after slaughter was very high (97.07%). Both the amount of *E. coli* contamination and the positive ratio of diarrheagenic *E. coli* in large-sized slaughterhouses (7.50–13.33 CFU/cm^2^, 3.44%) were lower than those in small- or medium-sized slaughterhouses (74.99–133.35 CFU/cm^2^, 5.71%). Combined with the current status of sanitary control in slaughterhouses, we determined that pre-cooling treatment significantly reduced *E. coli* and diarrheagenic *E. coli* in pork after slaughter, while microbiological testing reduced *E. coli*. Based on our monitoring data, China urgently needs to establish relevant standards to better control microbial contamination during pig slaughtering progress. This study provided a theoretical basis for the hygiene quality management of the pig slaughter industry in China.

## Introduction

In recent years, foodborne pathogenic infections have occurred frequently, which has a significant impact on human health and economy, and has become a major public health problem worldwide (1). Foodborne pathogens are the primary cause of foodborne diseases. Foodborne diseases caused by foodborne pathogens have been a major threat to food safety (2–4). With the increase in demand for fresh livestock and poultry products, an increasing amount of attention has been paid to food safety. Previous studies have shown that the number of outbreaks associated with fresh products increased from 0.7% in the 1970s to 6% in the 1990s in the United States (5, 6). According to the annual report statistics of the Rapid Alert System for Food and Feed (RASFF), the incidence of food notifications caused by pathogenic microorganisms has been fluctuating at a high level. Among the hazard category were concerned, pathogenic microorganisms were the most common hazard in meat and meat products in the years 2011–2015 (7). As a large country with high levels of pork production, pork accounts for 75% of the meat consumption in China. In recent years, domestic and foreign foodborne diseases caused by pathogenic microorganisms in pork have become very common (8–11). Quantitative microbiological risk assessment shows that slaughtering plays a significant role in the contamination of pig carcasses; therefore, it is very important to assess the microbial index in pork during the slaughter process in China (12–14).

According to reports, common pathogenic bacteria include: *Salmonella, Shigella*, diarrheagenic *E. coli, Campylobacter, Yersinia enterocolitica, Clostridium perfringens, Vibrio* and *Listeria monocytogenes*, etc (1). Studies have shown that in China, pork from pig slaughtering process and retail market was contaminated by various pathogenic bacteria in varying degrees, including *Campylobacter* (15), Shiga toxin–producing *E. coli* (11), *Listeria monocytogenes* (16), *Salmonella* (17), etc., which posed a great threat to people's life and health. Therefore, it is essential for the prevention and control of foodborne diseases by strengthening the public health surveillance of food-borne pathogens during animal production and formulating related hygiene specification and control standards in the process of “from farm to fork” (1).

*Escherichia coli* is a hygiene indicator bacteria for pig carcasses during slaughter; the level of *E. coli* contamination in the slaughtering process of live pigs has been found to be key to the health of the consumers. As a Gram-negative bacteria, most *E. coli* are generally non-pathogenic (18, 19). They are normally found living in human and animal intestines. Usually, *E. coli* is utilized as a hygiene indicator bacteria, reflecting the sanitary and safety status of food (20). A low amount of detected *E. coli* indicates that the food processing process is well-controlled and the product safety is good. In contrast, a high amount of detected *E. coli* indicates a poor hygiene level.

Presently, the risk of cross-contamination of various pathogenic microorganisms is also high during the slaughter of pigs. In this process, factors such as intestinal damage, fecal contamination, environment, equipment, or workers' hands may cause cross-contamination of pathogenic microorganisms. Therefore, the safety of the product cannot be guaranteed. Some serotypes of *E. coli*, known as diarrheagenic *E. coli*, including enteropathogenic *E.coli* (EPEC), Shiga toxin-producing *E. coli*/enterohemorrhagic *E.coli* (STEC/EHEC), *Shigella*/enteroinvasive *E.coli* (EIEC), enteroaggregative *E.coli* (EAEC), and enterotoxigenic *E.coli* (ETEC), can cause diarrhea, with food transmission as the main route of infection (21, 22). Diarrheagenic *E. coli* is the focus of pathogenic microorganism monitoring in livestock and poultry products in various countries. Monitoring data from the United States in 2015 showed that 5.0% of fresh pork was contaminated with diarrheagenic *E. coli* (23). Meanwhile, monitoring data from the European Union in 2017 showed that the contamination rate of diarrheagenic *E. coli* in fresh pork was 4.4% (24). Although the rate of diarrheagenic *E. coli* in pork is not high overall, once food safety issues are triggered, they can easily escalate and are more likely to be fatal to people with low immunity. Therefore, risk monitoring and assessment of diarrheagenic *E. coli* in pork products at the slaughter stage is very important.

In this study, we comprehensively analyzed microbial contamination of 2,013 samples from 71 pig slaughterhouses in 14 provinces of China from 2019 to 2020 to determine the current contamination status of *E. coli* and diarrheagenic *E. coli* in slaughtered pork in the country. This study provided technical support for the sanitary quality supervision and management of the pig slaughter industry in China.

## Materials and Methods

### Investigation of the Sanitary Conditions of Different Types of Slaughterhouses

Based on daily slaughter scales, in which the data calculation was in accordance with GB503175-2007 “Code for design of pig slaughtering and cutting rooms,” the slaughterhouses were divided into large-sized slaughterhouses, and small- or medium-sized slaughterhouses. The large-sized pig slaughter companies operate on daily slaughter scales exceeding 1,000 pigs, with slaughter and segmentation processing. The small- or medium-sized slaughterhouses have daily slaughter scales under 1,000 pigs. We conducted investigations into hygiene inspection and microbiological control of large-sized, and small- or medium-sized pig slaughter companies, in different regions, to determine the current hygiene control status of the industry.

### *Escherichia coli* Monitoring and Sampling

#### Sampling Points

We selected 71 pig slaughterhouses in 14 provinces of China from 2019 to 2020 [including ten provinces (Anhui, Shanxi, Shandong, Yunnan, Guangxi, Heilongjiang, Guangdong, Shanghai, Hubei, Xinjiang) in 2019 and eight provinces (Anhui, Shanxi, Shandong, Yunnan, Jiangsu, Sichuan, Henan, Jilin) in 2020; four of the provinces was sampled in both years]. There were 33 large slaughterhouses, and 38 small- or medium-sized slaughterhouses. The configuration of the slaughter cold chain and the development of microbiological testing in the slaughterhouses are shown in Table 1. In each province, at least two large-sized pig slaughter companies, and three small- or medium-sized slaughter companies, were selected for sampling.

**Table 1:** Sampled slaughterhouses.

**Number**	**Large slaughterhouses**	**Small- or medium-sized slaughterhouses**
Cold chain configurations for slaughter	26	17
Microbiological tests carried out	26	8
Sampling slaughterhouses	33	38

#### Sampling Period and Quantity

The period of May to October was selected for centralized sampling. According to the current situation of large slaughterhouses in China, the slaughtered pork is placed at 4°C for more than 6 h for precooling. In our study, at least 20 carcass surfaces swabbed before precooling, and 25 carcass surfaces swabbed after precooling, were collected from large slaughterhouses. At least 20 carcass surface swabbed samples were collected from small- or medium-sized slaughterhouses without precooling. A total of 1,074 carcass surfaces swabs were collected from large slaughterhouses, and 939 carcass surfaces swabs were collected from small- or medium-sized slaughterhouses. The total number of samples was 2013.

#### Sample Type and Transportation

Cotton swab (SWAB-10, Elabscience, USA) samples (involving pre-cooling) were collected from the surface of carcasses after slaughtering. Three sampling sites (belly, ham, and jowl) were selected on each sample, and 100 cm^2^ of cotton swab samples were applied to each sampling site. Environmental samples comprised the surface swabs of facilities and equipment, workers' hands, utensils, and the ground that directly or indirectly came into contact with the pig carcass during the slaughter process. The collected cotton swab samples were loaded into transport medium containing 10 mL phosphate-buffered saline, stored at low temperature, and transported to the laboratory for testing within 24–48 h.

### *Escherichia coli* and Diarrheagenic *E. coli* Detection

*Escherichia coli* counts were detected using commercial kits according to the industry standard SN/T 4547-2017, specifically using the Petrifilm *E. coli* test sheet (3M Petrifilm^TM^, USA), with reference to the instructions and the methods specified in the standard for detection. In short, 1 mL sample with appropriate dilution was added to the center of the Petrifilm plate, then slowly covering the upper film of the Petrifilm plate. After the sample was evenly covered the Petrifilm plate, place it horizontally at 36 ± 1°C and incubate for 24 ± 2 h. Record the number of blue colonies with gas on the Petrifilm plate and the corresponding dilution factor, expressed in colony forming units (CFU).

Samples from four randomly selected sampled provinces were tested for diarrheagenic *E. coli*. Diarrheagenic *E. coli* was identified using the multiplex polymerase chain reaction detection kit developed by our laboratory referring the study of Toma et al. (25).

### Data Analysis

The microbial contamination rate was analyzed by Excel software, and the amount of *E. coli* contamination was analyzed by MiniTab v.17 software (Minitab, Inc, State College, PA, USA). The contamination rate of pathogenic microorganisms was analyzed by the Pert distribution fitting in @RISK7 software (Palisade Corporation, New York, NY, USA), and the amount of *E. coli* contamination was analyzed by random distribution fitting. The function is obtained by random fitting. Monte Carlo simulation was performed using the Latin hypercube sampling method. Probability distributions were obtained from each time point in simulations of 10,000 iterations.

## Results

### Monitoring of *Escherichia coli* Contamination and Assessment of Diarrheagenic *E. coli* Exposure in Pork Produced in Different Types of Slaughterhouses

As a hygiene indicator, *E. coli* were detected and counted in pork after slaughter. Overall, there was a very high positive detection rate of *E. coli* in pork after slaughter in China, with an overall rate of 97.07% (1,954/2,013). Due to the fact that 55.26% (21/38, Table 1) small- or medium-sized slaughterhouses lack cold chain facilities, and considering the comparability of data between large-sized, and small- or medium-sized slaughterhouses, we detected and counted *E. coli* in pork before pre-cooling after slaughter in different types of slaughterhouses, and found that the isolation rate of *E. coli* was comparative between large-sized (97.30%) and small- or medium-sized slaughterhouses (96.81%).

**Figure 1:**
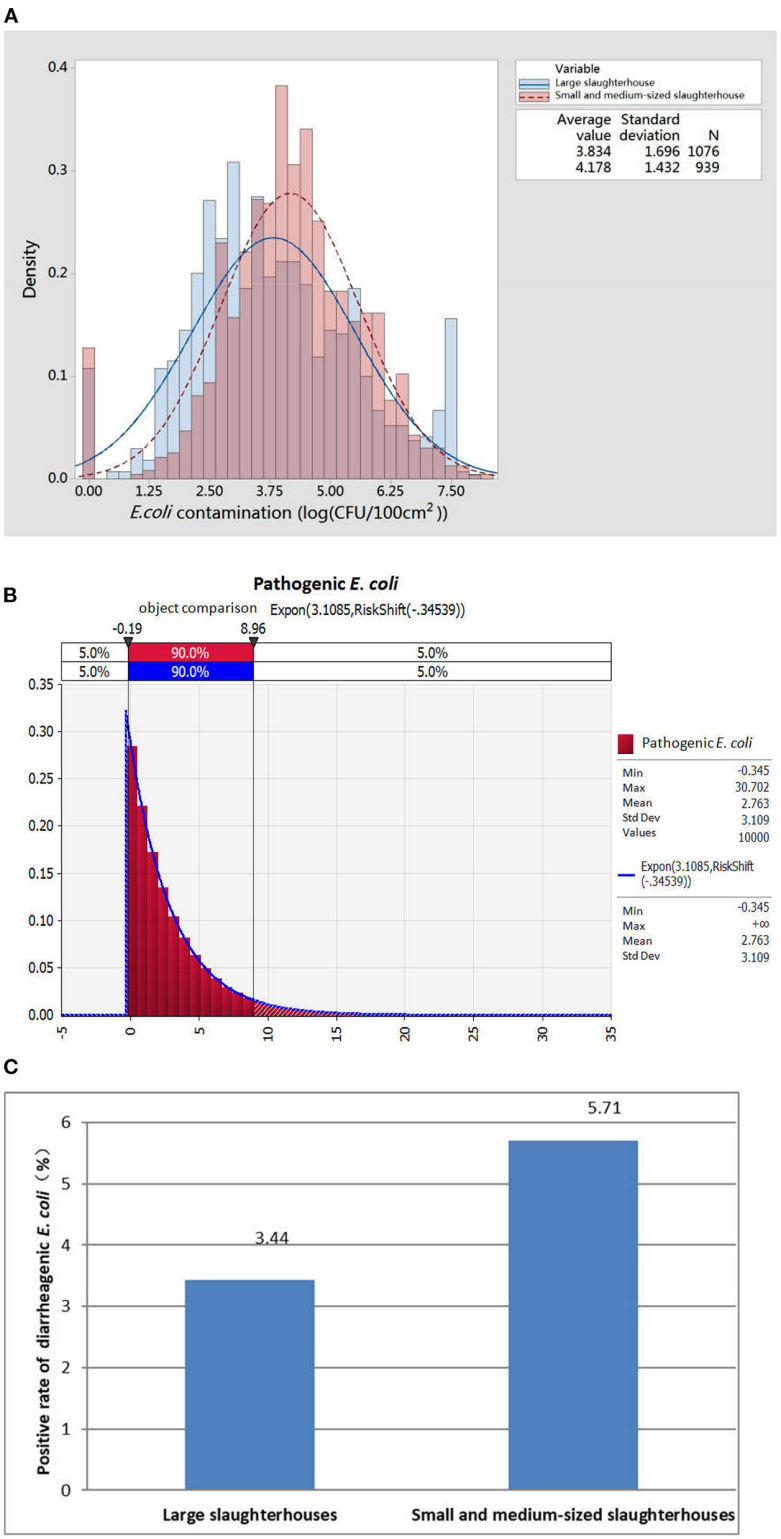
Monitoring of *Escherichia coli* and diarrheagenic *E. coli* contamination in pork produced in different types of slaughterhouses. **(A)** Distribution of *E. coli* contamination in pork from different types of slaughterhouses. **(B)** Exposure assessment results of diarrheagenic *E. coli* in pork during the slaughter process. **(C)** Exposure of diarrheagenic *E. coli* in pork produced in different types of slaughterhouses.

We separately compared the data distribution of *E. coli* detected in large-sized, and small- or medium-sized slaughterhouses. As shown in Figure 1A, the *E. coli* contamination of pork in large-scale slaughterhouses was generally lower than that in small- or medium-sized slaughterhouses. The highest frequency of contamination detected in small- or medium-sized slaughterhouses was 74.99 colony-forming units (CFU)/cm^2^-133.35 CFU/cm^2^, while the most frequently detected contamination in large-sized slaughterhouses was 7.50 CFU/cm^2^-13.33 CFU/cm^2^, which is exactly an order of magnitude difference. These results suggested that although the detection rate of *E. coli* in small- or medium-sized slaughterhouses was significantly different from that of large-sized slaughterhouses, the amount of *E. coli* contamination was still higher than that of large-sized slaughterhouses, indicating that overall hygiene control was poorer than that of large-sized slaughterhouses.

Next, the contamination rate of diarrheagenic *E. coli* in slaughtered pork was randomly distributed and fitted by @Risk RISK assessment software to determine the optimal function, and an exposure assessment was then carried out by Monte Carlo sampling (Figure 1B). The results showed that the contamination rate of diarrheagenic *E. coli* was mainly distributed in the range of 0–8.69% (mean value 2.76%). Meanwhile, the overall contamination rate of diarrheagenic *E. coli* in pork samples produced by large-sized slaughterhouses was 3.44% (32/931), and that of small- or medium-sized slaughterhouses was 5.71% (25/438) (Figure 1C). These results showed that the contamination of diarrheagenic *E. coli* in pork produced by small- or medium-sized slaughterhouses was greater than that of large slaughterhouses.

### Comparison of the Amount of *E. coli* Contamination, and Exposure Assessment of Diarrheagenic *E. coli* in Pre-cooled and Non-pre-cooled Pork

From a hygiene perspective, after pre-cooling cold fresh meat, the temperature and humidity changes of the carcass surface can deactivate the pathogenic microorganisms contaminated on the surface of the pork during the slaughter process, which better guarantees the quality and safety of the meat. Therefore, in this study, cotton swabbed samples from pork surfaces before and after pre-cooling were collected in some slaughterhouses with pre-cooling treatment for *E. coli* count analysis.

**Figure 2:**
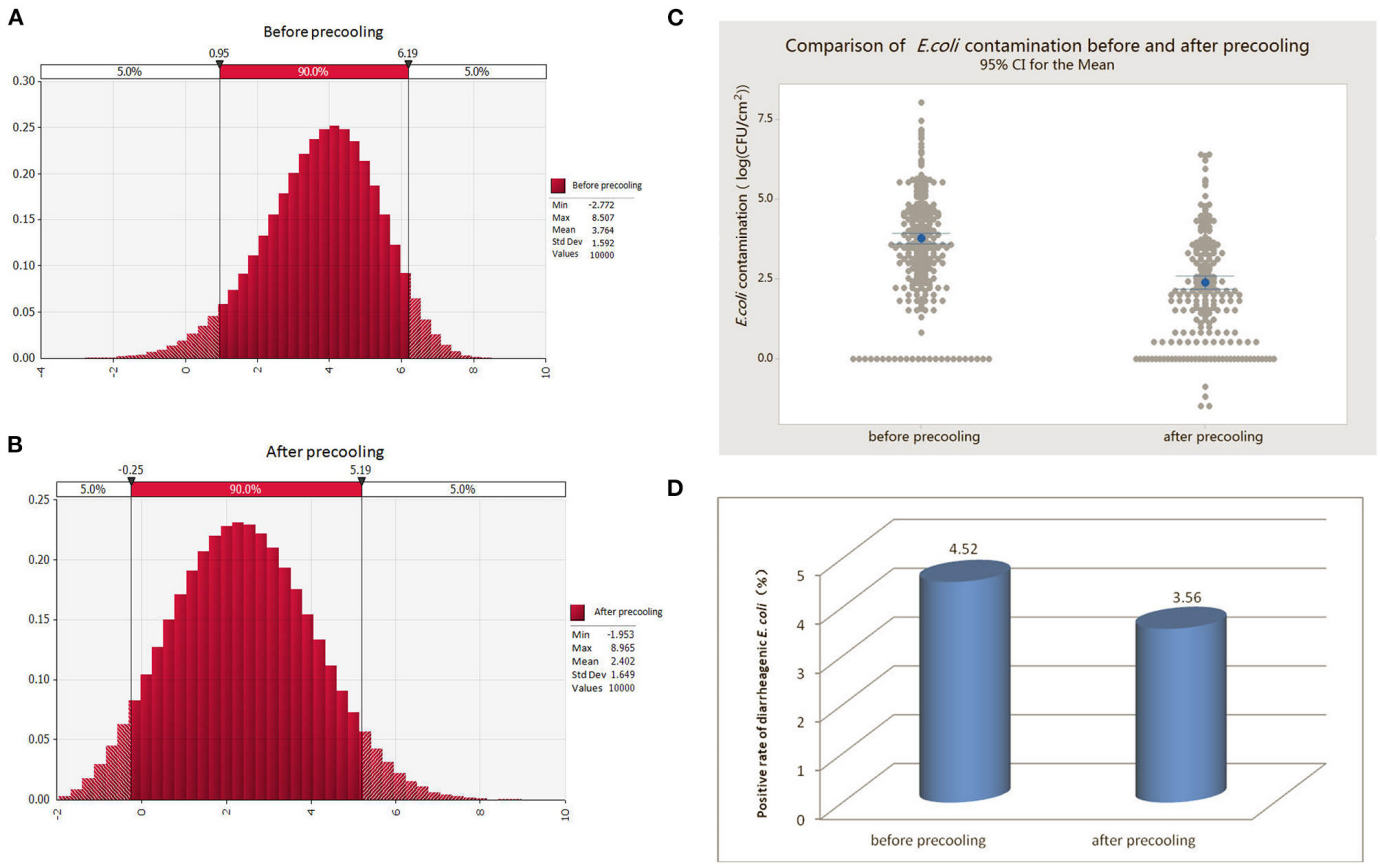
Comparison of the amount of *E. coli* and diarrheagenic *E. coli* contamination in pre-cooled and non-pre-cooled pork. **(A)** Probability distribution of *E. coli* contamination in pork before pre-cooling. **(B)** Probability distribution of *E. coli* contamination in pork after pre-cooling. **(C)** Comparison of *E. coli* contamination in pork before and after pre-cooling. **(D)** Exposure status of diarrheagenic *E. coli* contamination in pork before and after pre-cooling treatment.

By fitting the random probability distribution of *E. coli* contamination on the pork surface before and after pre-cooling (Figure 2A), the probability of 90% of the *E. coli* contamination before pre-cooling was 0.95–6.19 log (CFU/100 cm^2^) or 0.09 CFU/cm^2^-15,488.17 CFU/cm^2^; while the average was 3.76 log (CFU/100 cm^2^) or 57.54 CFU/cm^2^. After pre-cooling, the 90% probability of *E. coli* contamination was −0.25–5.19 log (CFU/100 cm^2^) or 0.005 CFU/cm^2^-1,548.82 CFU/cm^2^, and the average was 2.40 log (CFU/100 cm^2^) or 2.45 CFU/cm^2^ (Figure 2B). The results in Figure 2C show that pre-cooling treatment can greatly reduce the amount of *E. coli* in pork after slaughter. Considering that *E. coli* is a hygiene indicator, pre-cooling treatment may greatly reduce the contamination of pathogenic microorganisms in pork, thereby avoiding the chance of cross-contamination or infection in the subsequent circulation and consumption links.

In addition, we conducted exposure assessments of diarrheagenic *E. coli* in pork after slaughter before pre-cooling and after pre-cooling. The results showed that the overall contamination rate of diarrheagenic *E. coli* in pork samples was 4.52% (39/863) before pre-cooling and 3.56% (18/506) after pre-cooling (Figure 2D). In conclusion, pre-cooling significantly reduced the contamination of diarrheagenic *E. coli* in pork. Of note, we found that trend was similar to that of slaughterhouses, probably because large slaughterhouses generally performed pre-cooling treatment.

### Effects of Microbiological Testing on the *E. coli* Contamination of Pork After Slaughter

**Figure 3:**
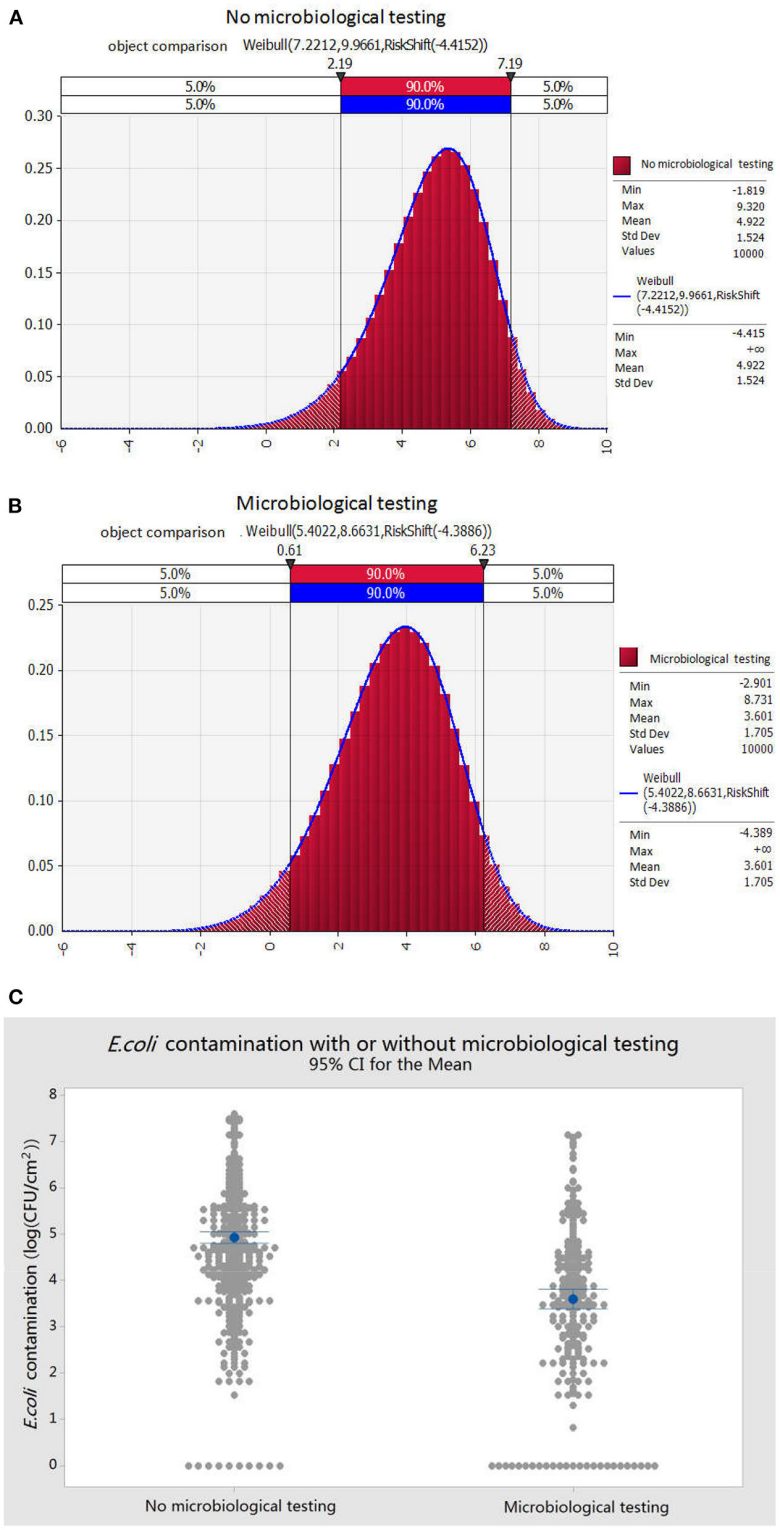
Effects of microbiological testing on *E. coli* contamination of pork after slaughter. **(A)** Contamination distribution of *E. coli* in pork during slaughter with microbiological detection. **(B)** Contamination distribution of *E. coli* in pork during slaughter without microbiological detection. **(C)** Comparison of *E. coli* contamination in pork during slaughter with or without microbiological detection.

Based on the preliminary investigation, we found that nearly half of the pig slaughterhouses carried out microbiological inspections. We compared and analyzed the contamination status of *E. coli* under the conditions of microbial inspection, not microbial detection in pork samples, before pre-cooling. By fitting the random probability distribution of *E. coli* contamination on the surface of pork after slaughter in two types of slaughterhouses, with and without microbial detection (Figure 3A), the probability of 90% of *E. coli* contamination without microbial detection was 2.19–7.19 log (CFU/100 cm^2^) or 1.55 CFU/cm^2^-15, 4881.66 CFU/cm^2^; while the average was 4.92 log (CFU/100 cm^2^) or 831.76 CFU/cm^2^. In contrast, the 90% probability of the contamination of *E. coli* with microbiological detection was 0.61–6.23 log (CFU/100cm^2^) or 0.04 CFU/cm^2^-16982.43 CFU/cm^2^; while the average was 3.60 log (CFU/100 cm^2^) or 39.82 CFU/cm^2^ (Figure 3B). The results in Figure 3C showed that microbial inspection played a significant role in promoting hygiene control in slaughterhouses, which could significantly reduce the amount of *E. coli* in pork after slaughter, thereby reducing the risk of potential pathogenic microorganisms in the slaughter process.

## Discussion

### *Escherichia coli* Contamination in Pig Slaughtering Chain in China Is Comparable to That of Developed Countries

In this study, we selected pig slaughterhouses in major pig producing provinces (respectively located in East China, South China, southwest China, central China, northwest China, and northeast China) for the monitoring of *E. coli*, which reflects the overall hygienic situation of pig slaughtering in China at the present stage. We found that the positive detection rate of *E. coli* in pork after slaughtering without precooling in China is very high, reaching 97.07% overall (1,954/2,013), which is similar to the 95.81% positive detection rate before evisceration in the United States (26). However, the United States pig slaughterhouses are equipped with cold chain facilities, after pre-cooling, the positive rate of *E. coli* decreased to 11.78% (26). In China, large-sized slaughterhouses with cold chain facilities account for ~80% of the total number of slaughtered pigs (Table 1). However, most small- or medium-sized slaughterhouses lack cold chain facilities (55.26%, 21/38, Table 1); therefore, the pork carries a large amount of *E. coli*, which enters the circulation. In the sales link, this undoubtedly increases the risk of food safety incidents. In addition, the total amount of *E. coli* contamination in pork after precooling in China was 2.45 CFU/cm^2^, which was even better than the monitoring data of 4.67 CFU/cm^2^ in the United States in 2011 (26). In addition, the exposure assessment results showed that overall, the contamination rate of diarrheagenic *E. coli* generally ranged between 0 and 8.69%, with an average of 2.76%, which is lower than the surveillance results for pathogenic diarrheagenic *E. coli* in the United States in 2015 (5%) and the European Union in 2017 (4.4%) (23, 24).

### Both Precooling and Microbiological Testing Reduced the Contamination of *E. coli* in Pork After Slaughtering

Research by H. Holly Wang et al. has shown that chilled meat and hot meat are the main pork types among Chinese consumers in the cities surveyed, accounting for 45 and 46%, respectively (27). The pre-cooling process can decrease the central temperature of the pork to ~4°C after slaughter. This process not only makes the pork taste better after the acid removal, but more importantly deactivates the microorganisms on the surface of the pork to ensure the safety of the meat. The average amount of *E. coli* contamination was 57.54 CFU/cm^2^ before precooling and 2.45 CFU/cm^2^ after precooling. Similarly, the contamination rate of diarrheagenic *E. coli* in pork before pre-cooling was 4.52%, which was reduced to 3.56% after pre-cooling. Thus, chilled pork will become a meat consumption trend in the future. GB/T 20551-2006 “Evaluating specification on the HACCP certification in the slaughter of livestock and poultry” (28) clearly states that the procedures for the inspection of Coliforms, *Salmonella spp*., and other harmful microorganisms should be established during the pig slaughter process and must meet the qualified requirements. We also found that the contamination of *E. coli* in pork produced in the slaughterhouses where microbiological inspection was carried out was greatly reduced. It is indicated that routine microbiological inspections in slaughterhouses had a positive effect on hygiene control and better guarantee product safety. However, in our investigations, we found that more than half of the pig slaughter companies did not carry out microbiological related testing and control, and even the companies that did this did not use a unified reference for monitoring technical specifications. Therefore, we recommend a standard operation of hygiene control in the slaughter process, effective implementation of microbial reduction measures (especially the development of microbiological inspections, etc.), establishment of a hygiene evaluation method for pig slaughter, and formulation of an evaluation index system, in order to improve slaughter hygiene, and the quality and safety of pork products.

### China Needs to Urgently Establish Control Standards of *E. coli* in the Pork Slaughtering Process

Developed countries such as Europe and the United States place great importance on the monitoring, assessment, and prevention and control of pathogenic microbial contamination in livestock and poultry products during the slaughter process, and have successively established a strict scientific regulatory and standards system (29, 30). However, in China, gaps remain in the standards for microbial control during the slaughter process. Because the monitoring results of *E. coli* in large-sized slaughterhouses are comparable to those in developed countries, and the microorganisms in the slaughter process can also be effectively controlled by measures such as pre-cooling, the control standards of *E. coli* in the pig slaughtering process must be urgently addressed. In fact, China is also taking a series of measures to improve the hygienic control of pig slaughtering process in recent years. For example, firstly, we promotes the standardization of pig slaughterhouses, which requires microbiological monitoring; secondly, we promotes the quality and safety risk monitoring of the slaughter process, which also stipulates the monitoring of common pathogens such as *E. coli* and *Salmonella*; thirdly, the overall bio-safety prevention and control measures of the slaughterhouse under the influence of African Swine fever (ASF) have been strengthened. It is believed that the development of standards for the detection of *E. coli* at the slaughtering stage can directly ensure the health and safety of the pork entering the market, and is also conducive to international standards.

In conclusion, the control of pork *E. coli* in large-sized slaughterhouses is better than that in small or medium-sized slaughterhouses in China, and *E. coli* contamination in the pig slaughtering chain in large-sized slaughterhouses after pre-cooling is comparable to that in developed countries. Therefore, corresponding standards are urgently needed to better control microbial pollution in pork during the slaughtering process.

## Data Availability Statement

The original contributions presented in the study are included in the article/supplementary material, further inquiries can be directed to the corresponding authors.

## Author Contributions

All authors listed have made a substantial, direct and intellectual contribution to the work, and approved it for publication.

## Funding

This work was funded by the National Key Research and Development Program of China (2018YFD0500505).

## Conflict of Interest

The authors declare that the research was conducted in the absence of any commercial or financial relationships that could be construed as a potential conflict of interest.

## Publisher's Note

All claims expressed in this article are solely those of the authors and do not necessarily represent those of their affiliated organizations, or those of the publisher, the editors and the reviewers. Any product that may be evaluated in this article, or claim that may be made by its manufacturer, is not guaranteed or endorsed by the publisher.
